# Spatial Attention Frustum: A 3D Object Detection Method Focusing on Occluded Objects

**DOI:** 10.3390/s22062366

**Published:** 2022-03-18

**Authors:** Xinglei He, Xiaohan Zhang, Yichun Wang, Hongzeng Ji, Xiuhui Duan, Fen Guo

**Affiliations:** School of Mechanical Engineering, Beijing Institute of Technology, Beijing 100081, China; thubiter@bit.edu.cn (X.H.); xhzhang810@bit.edu.cn (X.Z.); wych@bit.edu.cn (Y.W.); staypom@gmail.com (H.J.); dxhbit0250@163.com (X.D.)

**Keywords:** visual attention mechanism, occluded object detection, multi-sensor fusion, 3D object detection, autonomous vehicles

## Abstract

Achieving the accurate perception of occluded objects for autonomous vehicles is a challenging problem. Human vision can always quickly locate important object regions in complex external scenes, while other regions are only roughly analysed or ignored, defined as the visual attention mechanism. However, the perception system of autonomous vehicles cannot know which part of the point cloud is in the region of interest. Therefore, it is meaningful to explore how to use the visual attention mechanism in the perception system of autonomous driving. In this paper, we propose the model of the spatial attention frustum to solve object occlusion in 3D object detection. The spatial attention frustum can suppress unimportant features and allocate limited neural computing resources to critical parts of the scene, thereby providing greater relevance and easier processing for higher-level perceptual reasoning tasks. To ensure that our method maintains good reasoning ability when faced with occluded objects with only a partial structure, we propose a local feature aggregation module to capture more complex local features of the point cloud. Finally, we discuss the projection constraint relationship between the 3D bounding box and the 2D bounding box and propose a joint anchor box projection loss function, which will help to improve the overall performance of our method. The results of the KITTI dataset show that our proposed method can effectively improve the detection accuracy of occluded objects. Our method achieves 89.46%, 79.91% and 75.53% detection accuracy in the easy, moderate, and hard difficulty levels of the car category, and achieves a 6.97% performance improvement especially in the hard category with a high degree of occlusion. Our one-stage method does not need to rely on another refining stage, comparable to the accuracy of the two-stage method.

## 1. Introduction

With the surging demand for autonomous driving and robotics, 3D object detection has substantially progressed in recent years [1,2,3,4,5]. However, developing reliable autonomous driving is still a very challenging task. In the actual driving situation, dealing with occlusion problems in complex road conditions is closely related to the driving safety of autonomous vehicles. It is also a key factor restricting the performance of 3D object detection. Several existing 3D object detection methods have been explored to address these challenges. LiDAR-based Bird’s Eye View (BEV) methods do not suffer from scale and occlusion problems and have been widely used in various current 3D object detection methods [6]. The Ku projected 3D proposals onto the corresponding 2D feature maps for 3D object detection, fusing features from BEV maps and RGB images [7]. However, these methods suffer from losing critical 3D information during the projection process.

Some other methods extract features directly from the raw point cloud [8,9]. Although LiDAR sensors can provide accurate position information, it is challenging to rely on LiDAR sensors to identify the same objects of the previous and next frames in actual motion scenes. The image provided by the camera has rich semantic features and higher resolution, meaning that it can be used to quickly detect objects and implement tracking. Therefore, recent work fused multi-sensor information to obtain better detection performance. Chen proposed Multi-View 3D networks (MV3D), a sensory-fusion framework that takes both LIDAR point cloud and RGB images as the input and predicts oriented 3D bounding boxes [10]. Qi and Wang utilized a 2D detector to generate a 3D frustum and then regressed the parameters of the 3D box directly from the raw point cloud [11,12]. Compared to projection methods, multi-sensor fusion methods avoided losing information and took advantage of 2D image detectors. However, the performance of F-pointnet [11] was limited because the final estimate relied on too few front points, which could themselves be incorrectly segmented. Wang [12] gave up the estimation of foreground points and proposed FconvNet that tried to segment the frustum by aggregating point features into frustum features and estimating oriented boxes in 3D space. However, the same step size caused all point clouds in the frustum to have the same feature weights in feature extraction, which meant that the occluded objects lacked importance in the frustum. The detector does not know which part of the region should be of interest. The occluded objects in the scene could not be assigned sufficient feature weights, which resulted in features from unimportant regions in the feature map suppressing the features of the occluded objects.

**Figure 1:**
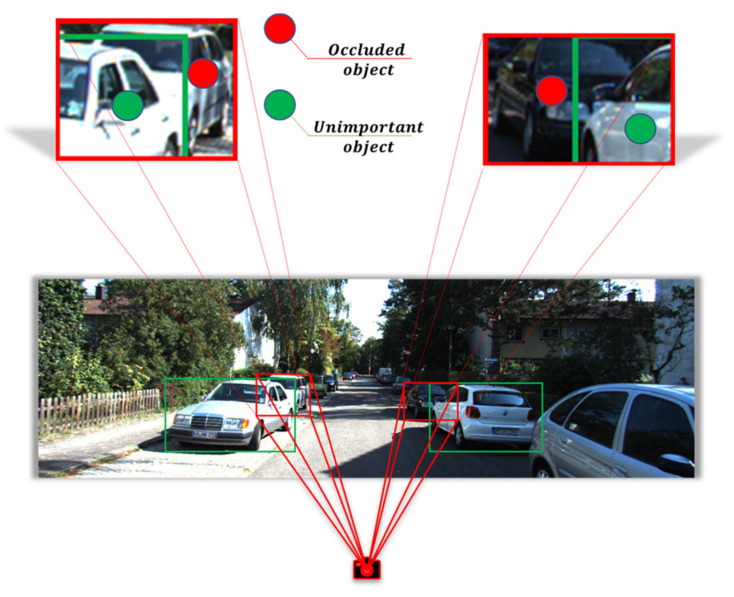
Common occlusion scene in autonomous driving.

**Figure 2:**
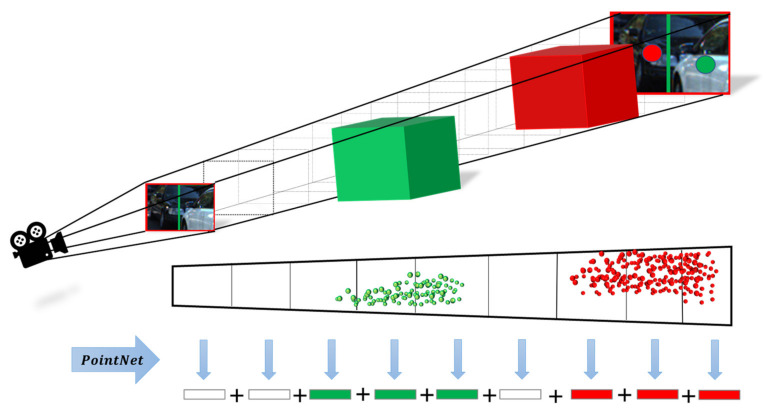
The frustum with the same length at each scale means that the unimportant point cloud and the attention point cloud cannot be effectively distinguished. The ‘+’ is concatenate operation.

This work is dedicated to studying the occlusion problem of 3D object detection. Figure 1 presents a typical occlusion scene. From our current viewpoint, the car in the red region is the occluded object of our interest, and the car in the green region is an unimportant object we do not care about. As shown in Figure 2, we generate a frustum from the 2D detection results of the red occluded object to detect it in 3D space. When the shorter-distance green object occludes the red occluded object, part of the point cloud of the green object appears in the frustum. The model cannot know in advance which part of the point cloud belongs to the occluded object, and the point cloud will be given the same importance because of the static segmentation method. As the distance increases, the point cloud density will become relatively sparse, which will cause the features of the essential region to be insignificant or even suppressed. This is not conducive to the detection of the occluded object.

To solve the limitations of F-pointnet and FconvNet, we tried to improve the feature saliency of the occluded object in the frustum further. When faced with a complex occluded scene, the human visual system quickly focuses on critical regions of the scene and suppresses unimportant information. Inspired by the visual attention mechanism, we proposed the spatial attention frustum (SAF). This study assumed that the occluded objects need to be partially visible rather than completely occluded, while the 2D detector has available performance. The spatial attention module can adaptively suppress unimportant features and allocate valuable neural computational resources to critical parts of the scene, which can be applied to faster and more efficient visual inference tasks. To ensure that the model still has good inference capability for objects with only partial structures, we proposed a local feature aggregation (LFA) module to capture more complex local features of point clouds and a joint anchor box projection loss (PL) function based on the projection constraint relationship between 3D bounding boxes and 2D bounding boxes. Our contributions can be summarized into four points:

- We proposed the SAF, which simulates the human visual attention mechanism to position occluded objects in autonomous driving scenes accurately. The SAF can adaptively suppress unimportant features and allocate valuable computational resources to the occluded objects in the frustum so that the features of the occluded objects can be more effectively represented in the limited feature space.
- Considering that the occluded objects usually have only the visible part of the point cloud, we proposed a point cloud local feature aggregation module to enhance the model’s ability to infer the whole from the local structure. The local feature aggregation module integrates more neighbourhood features, giving each point a larger perceptual field and allowing the model to learn more complex local features.
- We propose a joint anchor box PL function to obtain a more accurate boundary box prediction method by utilizing the projection constraint relationship between the 2D and 3D boxes. The experiment indicates that the joint anchor box PL function helps to improve the overall performance of the model.
- In the process of 3D object detection, our one-stage method can match the performance of the two-stage method without using refine stage, which makes our model more suitable for the autonomous driving scene in terms of real-time detection and the number of parameters.

## 2. Related Works

In this section, we briefly review the existing methods for 3D object detection, including image-based methods, LiDAR-based methods, and multi-sensor-based methods.

### 2.1. Image-Based 3D Object Detection Methods

There are several existing works on estimating the 3D bounding box from images. Chen proposed estimating 3D boxes using the geometry relations between 2D box edges and 3D box corners [13]. Zhang transformed 3D geometric information constraints into energy functions to correct the estimated 3D bounding boxes and faced the problem of insufficient depth information [14]. Brazil utilized the image grid and the location features of the 2D box centre to establish the relationship between the 2D box and the 3D box centre [15]. Weng converted the input image to the representation of a pseudo-LiDAR point cloud through monocular depth estimation and then used a 3D detection network trained end-to-end [16]. Wang used different optimization objects and decoders to estimate the foreground and background depth [17]. Dewi utilized generative adversarial networks (GAN) to enhance the image dataset and improve the recognition rate of the model [18]. Although these methods have demonstrated the feasibility of image-based methods, they are not effective in meeting the safety requirements of autonomous driving due to the lack of precise location information.

### 2.2. LiDAR-Based 3D Object Detection Methods

LiDAR can provide accurate location information, and there are many LiDAR-based methods to perform 3D object detection tasks. Zhou divided the point cloud into a certain number of voxels to classify and position regression [19]. Ye used a sparse convolutional middle extractor instead of 3D CNN [20]. Lang used PillarNet for feature extraction and transformed it into a presudo-2D image for bounding box regression [21]. Shi implemented foreground segmentation and rough prediction of bounding boxes and fused predictions and features to achieve accurate regression of the prediction box [22,23]. Ye proposed a hybrid voxel network, which used the attention mechanism to extract more fine-grained point cloud features to balance speed and accuracy [24]. Wang improved the prediction performance of the model by analysing the distribution of the point cloud to extract the features of the region of interest (ROI) [25]. Meyer used a fully convolutional network to predict a multimodal distribution over 3D boxes for each point, and then it efficiently fused these distributions to generate a prediction for each object [26]. Wang introduced domain adaption in migration learning to achieve cross-range adaptation and achieved better performance in the detection task for long-range objects [27].

### 2.3. Multi-Sensor-Based 3D Object Detection Methods

The image contains rich colour and semantic information, and the point cloud contains precise 3D geometric structure and depth information. Making full use of the advantages of the two types of information is beneficial for 3D object detection. Ku projected 3D proposals onto the corresponding 2D feature maps for 3D object detection, which improved the detection efficiency and reduced the difficulty of learning the 3D structure [7]. Xu fused RGB and the original 3D point cloud features [28]. Wang generated a series of frustums and aggregated multi-scale features [12]. Qi borrowed the voting ideas of VoteNet [29] and combined 2D votes on image 3D votes on the point cloud [30]. Zhu proposed a two-stage multimodal fusion network for 3D object detection, and they used pseudo-LiDAR points from stereo matching as a data augmentation method to densify the LiDAR point. The experiments on the KITTI dataset show that the proposed multi-stage fusion helps the network to learn better representations [31]. Vora proposed a sequential fusion method that projects LiDAR points into an image-only semantic segmentation network’s output and appends the class scores to each point [32].

## 3. Materials and Methods

In this section, we present our proposed method in detail. Section 3.1 presents the construction method of SAF. Section 3.2 presents the LFA module for the point cloud. Section 3.3 presents the joint 3D-2D anchor box PL function.

### 3.1. Spatial Attention Frustum (SAF) Module

**Figure 3:**
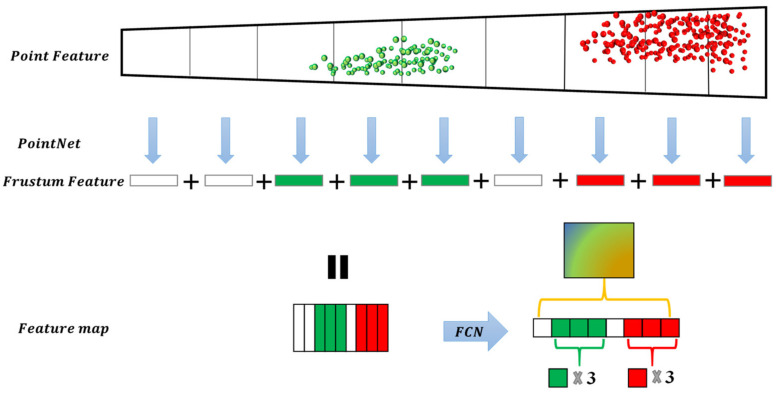
The feature vector of the unimportant object will seriously affect the expression of the feature vector of the object of interest in feature map. The ‘+’ is concatenate operation.

**Figure 4:**
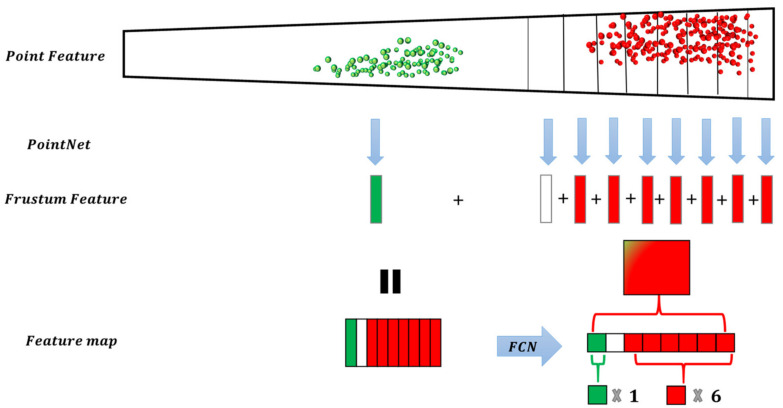
The frustum with spatial attention can improve the feature expression of the focused objects in the feature map. The ‘+’ is concatenate operation.

This study proposed a SAF module based on monocular depth estimation. The segmentation method for spatial attention is guided by object height, where the evaluation metrics of spatial attention are closely related to the distance estimation of objects. The F-pointnet indicated that finding the local point cloud corresponding to the suggested pixels in the 2D region can avoid traversing an extensive range of point clouds and improve detection efficiency. We hope to construct a model that resembles the mechanism of human visual attention to be able to observe the occluded objects more efficiently. Inspired by FconvNet, we further thought about the work of sliding the frustum. The point cloud density distribution follows the law of becoming sparser as the distance increases, and so the density of unimportant objects close to the occlusion scene is denser than that of the occluded objects in the distance. As shown in Figure 3, the fixed frustum sequence step size makes the unimportant point feature and the exciting point feature indistinguishable in the feature extraction process, which may cause computational costs to be wasted on detecting unimportant points, affecting the occluded object feature expression. The occluded object features’ weight is relatively small in the limited feature space, which leads to the feature expression not being significant in the subsequent process. The human visual attention mechanism ignores the unimportant object features and improves the weight of the occluded object features in the feature map. Therefore, we designed a frustum structure with spatial attention. As shown in Figure 4, with 2D region suggestions and camera parameters, the model can focus more on the occluded object features.

#### SAF Segmentation Method

We estimated a coarse distance for the model to focus on the features of the occluded object, while the exact regression of the 3D position was performed in the point cloud. Therefore, we chose a relatively lightweight approach to restore depth based on the principle of camera projection.

**Figure 5:**
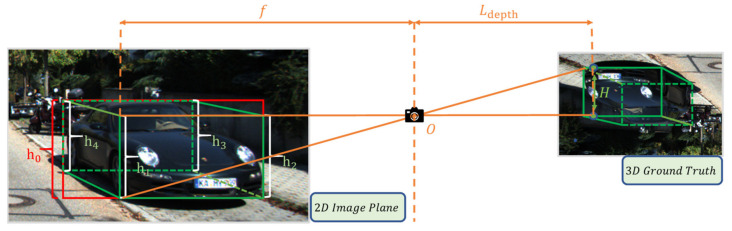
Projection relationship between ground truth and image.

As shown in Figure 5, H is the true height of the object in the 3D ground truth. The height H of each cuboid is fixed in the 3D real space, but the projection heights of the four vertical edges are different on the image plane projected by the camera. For H, we found the average height based on the height statistical characteristics of the dataset, and we used $\left(H=1.56\;m\right)$ for cars in the KITTI dataset. In the image plane, the more significant the height of the vertical edge projection, the closer the corresponding 3D spatial edge was to us. Therefore, we chose the side with the more significant vertical edge projection height to estimate the closest depth possible. For the relative depth $L_{depth}$ of each vertical edge of the box project on the image plane, we assumed that the camera was distortion-free, and solved for it according to Equation (1):

(1) $$L_{depth}=f\times \frac{H}{h_{i}}\;i\in \left(1,2,3,4\right)$$

where f is the focal length of the camera, $L_{depth}$ is the true distance from the optical centre O to the focused object, and $h_{1},\;h_{2},\;h_{3}\;$and $h_{4}\;$are the vertical projection heights of the cuboid of the target bounding box in the 3D space on the image plane.

Given the actual situation of the 2D detector, it would be complicated to calculate the vertical edge projection in 3D space on the image plane. Therefore, we ignored the regression performance of the 2D detector on the orientation angle of the 3D box and only obtained the class information and position information of the 2D box from the 2D detector $\left(x_{max},\;y_{max},x_{min},y_{min}\right)$. As shown in Figure 5, we were able to complete our rough depth estimation according to the results of the red box. We calculated the projection height according to Equation (2).

(2) $$h_{0}=y_{max}-y_{min}$$

The corresponding depth estimate was:

(3) $$L_{depth}=f\times \frac{H}{h_{0}}$$

The FconvNet verified that the multi-resolution frustum feature integration variant is effective. We referred to some of the original paper settings in the subsequent FCN module to facilitate feature alignment in subsequent operations. For the frustum of each region proposal, we proposed the following segmentation scheme. Table 1 shows the division scale and number of the frustum. First, the division scale is the division size of the frustum, which can also be interpreted as the resolution of the frustum-level features, and there are four levels of feature resolution, *T*, *T*/2, *T*/4, *T*/8. *T* is generally taken as a multiple of 8. The slice step of each sub-frustum is the length parameter along the axis of the apparent frustum, denoted as $L_{Ti}^{n}$. n is the resolution level of the division scale and takes the values of 1, 2, 3, 4. Its parameters are determined by the correction factor ω and $L_{pc}$. The correction coefficient ω is used to correct the distance estimation. $L_{pc}$ is the length of the extracted frustum in total. Num A is the number of frustums in the not interested region, and Num B is the number of frustums in the ROI. For a frustum of any scale, the frustum length $L_{T1}^{n}\;$and step length$\;d_{T1}^{n}$ of each not interested region segmentation are solved by Equation (4):

(4) $$\left\{\begin{array}{c} L_{T1}^{n}=\omega \times L_{depth}\\ d_{T1}^{n}=\omega \times L_{depth} \end{array}\right.$$

Each frustum length $L_{Ti}^{n}$ and step length $d_{Ti}^{n}$ of the frustums of ROI in the four scales are solved by Equations (5)–(8), respectively:

(5) $$\left\{\begin{array}{c} L_{Ti}^{1}=\frac{2\times \left(L_{pc}-\omega \times L_{depth}\right)}{T}\\ d_{Ti}^{1}=\frac{\left(L_{pc}-\omega \times L_{depth}\right)}{T} \end{array}\right.$$

(6) $$\left\{\begin{array}{c} L_{Ti}^{2}=\frac{2\times \left(L_{pc}-\omega \times L_{depth}\right)}{T/2}\\ d_{Ti}^{2}=\frac{\left(L_{pc}-\omega \times L_{depth}\right)}{T/2} \end{array}\right.$$

(7) $$\left\{\begin{array}{c} L_{Ti}^{3}=\frac{2\times \left(L_{pc}-\omega \times L_{depth}\right)}{T/4}\\ d_{Ti}^{3}=\frac{\left(L_{pc}-\omega \times L_{depth}\right)}{T/4} \end{array}\right.$$

(8) $$\left\{\begin{array}{c} L_{Ti}^{4}=\frac{2\times \left(L_{pc}-\omega \times L_{depth}\right)}{T/8}\\ d_{Ti}^{4}=\frac{\left(L_{pc}-\omega \times L_{depth}\right)}{T/8} \end{array}\right.$$

### 3.2. Local Feature Aggregation (LFA) Module

The occluded object is usually only a part of the point cloud visible with LiDAR, and the lack of some features will increase the difficulty of recognition. Enhancing the understanding of the local structure of the object is crucial, because sometimes it is needed to infer the whole object position from a smaller number of local point clouds. We thought that each point should have a larger sensory field in the point sampling stage and ensure sampling efficiency. The current common point cloud sampling methods were analysed and compared in the selection of sampling methods. Farthest point sampling (FPS) was considered first to ensure good coverage of the sampled points. However, when dealing with large-scale scenes of point clouds, the complexity of the squared calculation will result in the more unsatisfactory real-time performance of the model. The grid sampling method uses grid points to discrete 3D space, and then samples each grid point and controls the spacing between points by controlling the size of the grid points, but its uniformity is not as good as the FPS method. The sampling method based on the point cloud curvature shape is stable, but the long curvature computation time causes it to be unsuitable for large-scale datasets. Random sampling (*RS*) has the most efficient constant computational complexity and good dataset scalability but will inevitably result in the loss of some useful information, which will adversely affect the feature representation of the model.

**Figure 6:**
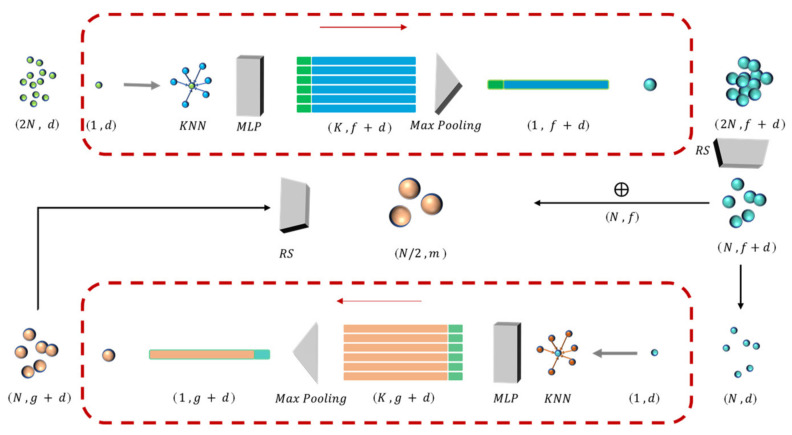
The LFA module.

This study selected the *RS* algorithm, which allows the model to work well when facing datasets of any size. Inspired by the work of RandLA-Net and pointnet [33,34], we defined an LFA module to increase the receptive field of each point. The LFA module is based on the K nearest neighbour (*KNN*) algorithm to find the nearest *K* neighbour points. Figure 6 illustrates the feature aggregation and the down-sampling process of the LFA module. The red dashed box shows the feature aggregation process for the sampled points, and the number of point clouds after each *RS* operation is reduced to half of the original number.

For any one of the frustums, assume that it contains M local points, which are represented in the camera coordinate system as $P_{camera}={\left(x_{i},y_{i},z_{i}\right)}_{camera}$. Instead of the coordinates in the camera coordinate system, we used the relative coordinate ${\left(x_{c},y_{c},z_{c}\right)}_{frustum}$ to the centre of the current frustum, which is calculated as $P_{i}=\left(x_{i}-x_{c},y_{i}-y_{c},z_{i}-z_{c}\right)$. The *KNN* algorithm can find the nearest *K* neighbourhood points $\left\{p_{i}^{1},p_{i}^{2},p_{i}^{3},\ldots ,p_{i}^{k}\right\}$ in the Euclidean space for each point $P_{i}$, the Euclidean space coordinate characteristic of each point is d, and the point feature of each neighbor point is $\left\{f_{i}^{1},f_{i}^{2},f_{i}^{3},\ldots ,f_{i}^{k}\right\}$. Then, the local map structure position encoding is performed, and the 3D coordinate of $P_{i}$. A multilayer perceptron (MLP) maps features to high-dimensional space, connects them to the original neighbouring point features, and pools them. The output result is used as a new point feature. The vector mapping relationship of neighbour features is as follows:

(9) $$G\left(p_{i},p_{i}^{k}\right)=MLP\left(p_{i}^{k}⊕(p_{i}-p_{i}^{k}\right)⊕ǁp_{i}-p_{i}^{k}ǁ⊕p_{i})$$

The new point features are as follows:

(10) $$f_{i}=MaxPool\langle G\left(p_{i},p_{i}^{k}\right)\rangle ⊕MLP\left(p_{i}\right)\;i\in \left\{1,2,\ldots ,n\right\},\;k\in \left\{1,2,\ldots ,m\right\}$$

In Equations (9) and (10), *MLP* represents the multi-layer perceptron, MaxPool represents maximum pooling, $p_{i}$ is the coordinate of the selected point, $p_{i}^{k}$ is the coordinate of the neighbour point, ⊕ is concatenate operation, $p_{i}-p_{i}^{k}$ is the relative coordinate, and $ǁp_{i}-p_{i}^{k}ǁ$ is the Euclidean distance. The *KNN* algorithm ensures that the neighbouring points can still be extracted in the sparse region of the point cloud. After two down-sampling processes of the aggregated local features, the sampled points can be considered to have a larger perceptual field. The local graph structure embeds the coordinates of all neighbouring points and efficiently learns the complex local structure to retain more local features.

### 3.3. Feature Extractor and Fully Convolutional Network (FCN)

As with FconvNet, we used the pointnet with weight sharing for parallel processing and aggregated point feature into the frustum feature. The pointnet module consists of three MLP layers and one Max Pooling layer. Pointnet with T numbers of shared weights aggregates the features of T numbers of a subfrustum into a frustum level feature vector. The T feature vectors are combined into a 2D feature map F of size T×d, used as the input of a subsequent FCN. The FCN contains four convolution layers and three deconvolution layers. Each convolutional layer is followed by batch normalization and ReLU nonlinearity. Except for the first convolutional layer, each convolution block uses stride-2 convolution to down-sample the 2D feature maps, so the output feature map of the convolutional block in FCN has a 2-fold lower resolution in the frustum dimension. When the scale is T/2, the feature map is compatible with its corresponding one in FCN. To maintain the integrity of the FCN, we concatenate the feature vectors extracted in the T scale down-sampling process and the feature vectors of the T/2 scale and use a fusion convolution layer to keep the size constant. The feature map output by each convolution block uses the corresponding deconvolution block for up-sampling. It concatenates all deconvolution outputs together with the feature size. Our detection header includes parts of CLS and REG.

### 3.4. Projection Loss Function

To fully exploit the excellent performance of the 2D detector, inspired by [31], we proposed a 3D-2D coupled loss function in the regression stage to obtain a more accurate 3D box estimate. The ideal 2D bounding box corresponds to the projection of the 3D bounding box in the image plane. Therefore, it is necessary to make full use of the constraints of the 2D bounding box on the 3D bounding box in the regression process of the 3D bounding box. The ground truth of the 3D bounding box is represented as $\left\{x_{g},y_{g},z_{g},l_{g},w_{g},h_{g},\theta_{g}\right\}$ in the LiDAR coordinate system, where $\left\{x_{g},y_{g},z_{g}\right\}$ denote coordinates of box centre,$\;\left\{l_{g},w_{g},h_{g}\right\}$ denote three side lengths of the box, and $\left\{\theta_{g}\right\}$ is the object orientation from the BEV. The 2D bounding box is represented as $\left\{x_{g},y_{g},l_{g},h_{g}\right\}$, where $\left\{x_{g}^{\prime },y_{g}^{\prime }\right\}\;$is the 2D bounding box centre and $\left\{l_{g}^{\prime },h_{g}^{\prime }\right\}$ is the 2D bounding box size. The projection relationship from a point x in the Velodyne LiDAR coordinate system to image coordinate y is as follows:

(11) $$y=P_{rect}^{i}R_{rect}^{0}T_{velo}^{cam}x$$

In Equation (11), x is the homogeneous coordinate form of the point cloud, $P_{rect}^{i}$ is a 3×4 projection correction matrix containing camera parameters, $R_{rect}^{\left(0\right)}$ is a 4×4 rectifying rotation matrix of the reference camera, and $T_{velo}^{cam}$ is the external parameter matrix of the LiDAR and camera obtained by calibration, including the rotation matrix$\;R_{3\times 3}$ and translation matrix T, as follows:

(12) $$T_{velo}^{cam}=\left|\begin{array}{cc} R_{3\times 3} & T\\ 0 & 1 \end{array}\right|$$

We followed existing study [12] for anchor boxes generation. For any one of them represented as $\left\{x_{p},y_{p},z_{p},l_{p},w_{p},h_{p},\theta_{p}\right\}$, the centre offsets $\left\{\Delta x,\Delta y,\Delta z\right\}$ predefined size offsets $\left\{\Delta l,\Delta w,\Delta h\right\}$ and the orientation $\left\{\Delta \theta \right\}$ were computed. For the regression for projection of 3D bounding box, we projected regressed 3D anchors onto images to generate 2D anchors of size $\left\{x_{p}^{\prime },y_{p}^{\prime },l_{p}^{\prime },h_{p}^{\prime }\right\}$, and computed 2D centre offsets $\left\{\Delta x^{\prime },\Delta y^{\prime }\right\}$ and size offsets $\left\{\Delta l^{\prime },\Delta \;h\;\prime \right\}$. We calculated the offset by using Equation (13).

(13) $$\Delta x=x_{g}-x_{p},\Delta y=y_{g}-y_{p},\Delta z=z_{g}-z_{p}\;\Delta l=log\frac{l_{g}}{l_{p}},\Delta h=log\frac{h_{g}}{h_{p}},\Delta w=log\frac{w_{g}}{w_{p}}\;\Delta \theta =sin\left(\theta_{g}-\theta_{p}\right)\;\Delta x^{\prime }=x_{g}^{\prime }-x_{p}^{\prime },\Delta y^{\prime }=y_{g}^{\prime }-y_{p}^{\prime }\;\Delta l^{\prime }=log\frac{l_{g}^{\prime }}{l_{p}^{\prime }},\Delta h^{\prime }=log\frac{h_{g}^{\prime }}{h_{p}^{\prime }}$$

The regression loss function is as follows:

(14) $$Loss=L_{cls,3D}+\gamma (\underset{res\in \left\{x,y,z,l,w,h,\theta \right\}}{\sum }L_{smooth-L1}\left(\Delta res\right)\;\;\;\;\;\;\;\;\;\;+\mu \underset{pro\in \left\{x,y,l,h\right\}}{\sum }L_{smooth-L1}\left(\Delta pro\right)+\varphi L_{coner})$$

The regression loss (Equation (14)) is based on the Euclidean distance and smooth-L1 regression loss for offsets of size and angle, including Δres and Δpro. γ, μ, and φ are loss coefficients. The focal loss [35] is used to calculate point segmentation loss $L_{cls,3D}$ to handle the class imbalance issue:

(15) $$L_{cls,3D}\left(P\right)=-\sigma {\left(1-P\right)}^{\tau }log\left(P\right)\;$$

where

$$P=\left\{\begin{array}{c} \;\;\;\;\;\;\;p,\;\;for\;foreground\;points,\\ 1-p,\;\;otherwise. \end{array}\right.$$

where p is the predicted foreground probability of a single 3D point, and we use a corner loss $L_{coner}$ [11] to regularize box regression of all parameters.

## 4. Experiments

This section evaluates our proposed 3D object detector on the public KITTI benchmark [36], and our method will be compared with previous methods in the 3D object detection task. Section 4.1 introduces our dataset and some experimental details. Section 4.2 provides a full ablation experimental study and analysis of the various components of the model. Section 4.3 shows the visualization of the results of the 3D detection model on the KITTI dataset. Section 4.4 shows the results of comparison with other methods.

### 4.1. Dataset

The KITTI dataset is one of the most popular autonomous driving datasets available. As the ground truth of the test set is unavailable, we refer to existing work [12] for dataset division and evaluation approaches. We follow the convention and use the car category containing the most training examples for the ablation study. The official 3D IOU evaluation metric for cars, pedestrians and cyclists are 0.7, 0.5 and 0.5, respectively. The mean average precision (mAP) is our evaluation metric following the official evaluation protocol. The KITTI evaluates 3D object detection performance using the PASCAL criteria also used for 2D object detection. Distant objects are thus filtered based on their bounding box height in the image plane, and the three difficulty categories are as follows:

For the easy category, the minimum bounding box height is 40 Px and the max truncation is 15%; for the moderate category, the minimum bounding box height is 25 Px and the max truncation is 30%; for the hard category, the minimum bounding box height is 25 Px and the max truncation is 50%. More details about difficulties are defined in Table 2.

### 4.2. Implementation Details

We used the 2D detection results provided by the FconvNet. For the LiDAR backbone network, we set the depth range in KITTI to (0, 75) meters. We performed two down-sampling operations on the 3D space corresponding to each region proposal, each at half the size of the previous one, for a final number of sampled point clouds of 1024. To prepare the positive and negative training samples, we scaled down the ground truth box by 0.5, counted the anchor box as the foreground box and counted the others as the background, and ignored the anchor box centred between the reduced box and the ground truth. We also performed random flipping and shifting to these points, similar to the FconvNet. We trained our model with a mini-batch size 32 for 60 epochs on one NVIDIA Quadro M6000 GPU. We used the ADAM optimizer with an initial learning rate of 0.001, and the weight decayed to 0.001 every 10 epochs. For the car category, the frustum resolution was four groups. We present the number of groups of four frustums in training for cars category as $\left\{240,120,60,30\right\}$. We kept σ = 0.25 and τ = 2, in accordance with the original paper on regression loss, and we set loss weight μ = 0.1. In the evaluation phase, we used an NMS module with a 3D IOU threshold of 0.1 to reduce redundancy. The final 3D detection score was calculated from the 2D detector and the predicted 3D bounding box scores.

### 4.3. Ablation Study

**Figure 7:**
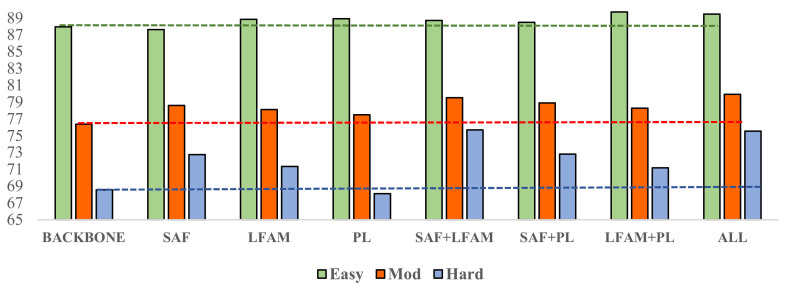
The effect of different modules on performance improvement.

This section verifies components and variants proposed by conducting ablation studies on the validation split of KITTI. We used the official training and validation splits and accumulate the evaluation results over the whole training set. We followed the convention and used the car category that contains the most training examples. While [9] proposed a refining stage to modify the estimation of the bounding box, the refining stage will destroy the integrity of the model and slow down the detection speed. Therefore, we used the structure part that does not include the final refine process as the backbone and used the results reproduced in the data above to set the division standard as the baseline. The ablation study results are shown in Table 3 and Figure 7.

#### 4.3.1. Effects of the SAF Module

Table 3 shows the effect of the SAF module. In the three difficulty levels of easy, moderate, and hard in the 3D detection results, the SAF contributed −0.33%, 2.22%, and 4.18% to the overall accuracy improvement, and the SAF plays a crucial role in improving the detection accuracy of the moderate and hard difficulty levels. For the accuracy improvement effect of the SAF at each difficulty level, the results of the easy difficulty level demonstrate that the detection accuracy decreased compared with the baseline results. We speculate that in the standard of the KITTI dataset, the occlusion level in the easy difficulty level is fully visible. There are almost no unimportant points in this case so we can think that the features extracted by the model are based on the objects we are interested in. The biased depth estimation may lose some features when detecting obvious objects, so SAF does not perform easy difficulty-level detection well. However, in the moderate difficulty level and the hard difficulty level, the occlusion will make our model focus on the occluded object and suppress the feature of the not interesting object to make the feature of ROI more prominent, which is also the key to improving the performance at the moderate difficulty level significantly and the hard difficulty level compared with the backbone.

We further analyse the test results of SAF combined with other modules alone. As can be seen from Table 3, the structure with the combination of SAF and LFA achieves the highest accuracy and exceeds the sum of the accuracy of the two modules alone regarding the difficult level, which indicates that the two modules can promote each other regarding the difficult level. The LFA better retains the local structure features. It is crucial to learn these local features for the objects of interest for whom only part of the point cloud is visible. Similarly, most detection accuracy improvement is due to combining the two modules at the moderate difficulty level. The effect of the two modules at the easy difficulty level is improved, indicating that the introduction of LFA makes up for the loss of SAF performance to a certain extent, but the overall improvement is still limited.

#### 4.3.2. Effects of the LFA Module

As shown in Table 3, applying the LFA module brings a 0.89%, 1.74%, and 2.77% gain in 3D detection at the easy, moderate, and hard difficulty levels, respectively. The results display the 3D detection performance of the car class on the validation split of KITTI. The local features aggregation module improves the overall performance of the model, which has a good effect on the improvement of the detection performance of the three difficulty levels. As shown in Table 2, objects at the easy level are fully visible. The point cloud’s general information is more abundant, enabling the model to make more accurate inferences without relying too much on neighbour point features. Therefore, the performance of the LFA module at the easy level is only improved by 0.89%. However, LFA increased the effect on objects with more severe occlusion at the moderate and hard difficulty levels. The experimental results show that LFA can effectively aggregate the features of neighbouring points and infer the overall structure of the occluded object for which only a local visible point cloud is available. Finally, considering the differential configuration of cars, an efficient random sampling method allows the model to maintain good usability when dealing with datasets of any size.

#### 4.3.3. Effects of the PL Loss Function

The impact of the 3D-2D projection loss module on performance is shown in Table 3, where the module contributes 0.96%, 1.11%, and −0.47% to the improved detection accuracy. In the performance improvement at the easy difficulty level, a single PL module has the best performance, and the joint action with SAF can make up for the error effect caused by the depth estimation. At the hard difficulty level, the introduction of the PL module leads to a decrease in performance. When the detection object is far away, the slight change in the projection coordinate may cause a significant change in the predicted 3Dbox, so the performance of the PL module is better over shorter distances than over longer distances. In subsequent experimental parameter settings, the accuracy impact of the three difficulty levels will be set within an acceptable range. Considering the overall performance of the model, we believe that it is necessary to utilize the constraint relation of 3D-2D effects on the projection.

#### 4.3.4. Effects of Feature Extractor

In the structure of the feature extraction from the point cloud, we tested and compared the impact of pointnet [34] and pointnet++ [37] on performance. Before the test, we thought that the pointnet++ incorporating local features would show better performance, but we did not obtain the expected accuracy improvement in actual verification. We believe that the reasons for this result are as follows: our model’s local feature enhancement module already contains part of the local information, which causes the sampling module of pointnet++ to be unable to play many roles. In some sparse point cloud regions, the radius-based sampling method of pointnet++ may lead to insufficient sampling points in this region and loss of information. Finally, since the sampling method of pointnet++ is based on the FPS method, considering the number of model parameters and good dataset scalability, we abandon the pointnet++ method and adopt the relatively lightweight pointnet.

### 4.4. Qualitative Results

**Figure 8:**
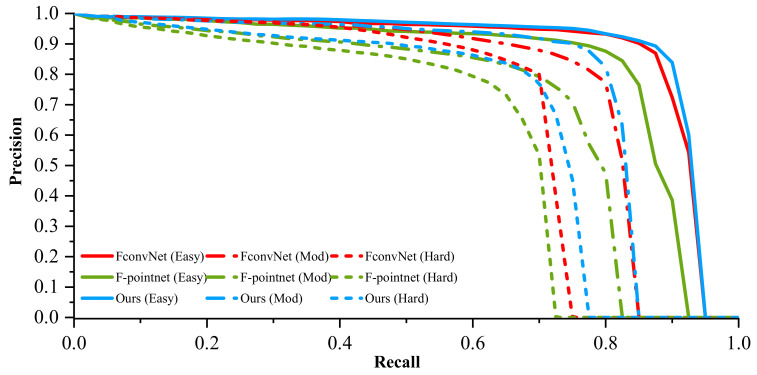
The precision-recall curves for car 3D detection at all levels of difficulty.

**Figure 9:**
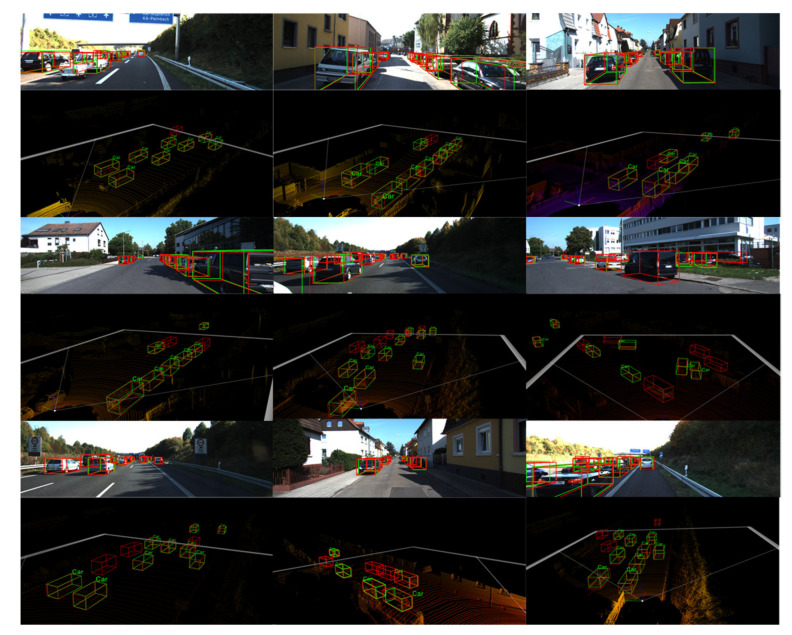
Qualitative results on the KITTI.

The precision–recall (PR) curve is useful for measuring whether the method is good enough for all the positive and negative samples, and it can be calculated as $Precision=TP/\left(TP+FP\right)$ and $Recall=TP/\left(TP+FN\right)$. Here, TP is the true positive, FP is the false positive, and FN is the false negative. Figure 8 shows the PR curves for car 3D detection at three difficulty levels. Our method is always the best when compared with F-pointnet. Compared with the FconvNet with a refining stage, our one-stage method also has good model performance. We show the proposed detector’s qualitative 3D object detection results on the KITTI benchmark in Figure 9. The upper part is the image, and the lower part is the visualization of the 3D point cloud. We use red bounding boxes to represent the predicted bounding boxes and green bounding boxes representing the ground truth-bound boxes for better visual comparison. As shown in Figure 8, the 3D box of the occluded object is estimated accurately, which shows that our method has good detection performance for long and highly occluded cars.

## 5. Discussion

As shown in Table 4, we compared the performance of the KITTI validation set with methods that also rely on the 2D detector. Several methods used the same results provided by the 2D detector and routinely evaluate the most numerous three categories to be fair. The earliest work is F-pointnet. To be precise, this is a three-stage approach. After the completion of the 3D instance segmentation process, it was necessary to train a transformation network to return the residuals between the coordinate origin and the centre of the real object. Experiments show that this stage has a critical impact on performance. As an improvement, FconvNet removed the T-Net structure but added a refinement stage to improve the detection accuracy. The ablation experiments showed that this strategy significantly improved the accuracy of object detection. However, it is also a two-stage method. To facilitate performance comparison, we referred to some settings of the FconvNet and repeated the tests on the baseline dataset. We used a one-stage network as the backbone and separately listed the two-stage experiment results that joined the refine stage. We performed a four-fold cross-validation and averaged the experimental results. The algorithm runtime is very important for self-driving vehicles. As the algorithm runtime is affected by the computational speed of the hardware device, in order to be fair, we implemented FconvNet and our proposed model on the same device, because the mechanisms of these models all work based on 2D detectors. Table 4 compared the above methods and achieved state-of-the-art performance at easy and moderate difficulty levels for the car category. The test results for the hard difficulty level show that our one-stage method does not require an additional refinement stage and has a performance comparable to that of the two-stage method. The one-stage method has superior deployment value in subsequent applications because real-time and small parameters are critical for autonomous driving. Moreover, our model is reduced by almost half of the parameters due to the absence of the refinement stage. Table 5 and Table 6 compare the results of the above methods on the pedestrian and cyclist categories, and the proposed method exceeds the F-pointnet and the FconvNet without the refinement stage in terms of accuracy, which indicates the effectiveness of our proposed model. However, unlike the performance of the vehicle category, the proposed method does not perform as well as the FconvNet model with the refinement stage for both the pedestrian and cyclist categories. There may be two reasons for this: (1) the value of H in Equation (1) needs to be further optimized; and (2) the number of pedestrians and cyclists in the dataset is much smaller than the number of vehicle categories, which means that there is an imbalance problem in the dataset, and our proposed method provides limited improvement in the detection accuracy for the pedestrian and cyclist categories. Finally, although FconvNet with a refinement stage achieved the highest detection accuracy, it also resulted in the longest runtime. The advantage of our proposed model is that it achieves a compromise between accuracy and runtime.

Table 7 compared the existing methods on the KITTI validation set. By comparing with [7,11,12,19,20,22,38,39,40], we obtained significant accuracy improvements at moderate and difficult difficulties, which indicates that our method effectively improves the detection performance of obscured objects and has comparable performance to the two-stage method. Moreover, it is necessary to utilize RGB and LiDAR multimodal information to improve the overall performance of the model compared to the LiDAR-based methods. There is also interest in exploring the effect of the occlusion percentage on detection accuracy. In fact, as shown in Table 2, three occlusion levels are provided in the official KITTI dataset and expressed by the percentage of occlusion, and the results of the three difficulty categories in Table 4, Table 5, Table 6 and Table 7 reflect the impact of the percentage of occlusion on the detection accuracy. Conservatively, the maximum percentage of partial occlusion that can be detected by the proposed method is 50%. However, as shown in Figure 9, some occlusion percentages above 50% were not officially labelled, but were still successfully detected by our model. Therefore, further research on this issue relies on further high-precision labelling of the dataset by KITTI.

## 6. Conclusions

In this paper, we propose a 3D object detection method with spatial attention, which improves the detection performance of occluded objects. The SAF module achieves a significant representation of the features of the occluded objects in a limited feature space. The LFA module enhances the understanding of the local structure of the occluded object. It allows better inference of the overall structure of the obscured object from a small number of locally visible point clouds when confronted with only the visible part of the object. We explored the feasibility of fully exploiting the 3D-2D constraint relationship, and the experimental results show that the joint 3D-2D anchor box projection loss helps to improve the overall performance of the model. Finally, compared to the baseline, our method significantly improves the detection accuracy of obscured objects without additional stages, suitable for real-time and parametric autonomous driving scenarios. The present limitation of this work is that we have assumed for now that the 2D region proposals are accurate enough. Therefore, when the 2D detector fails to detect the occluded object on the image, it also fails to generate a frustum for the occluded object. In addition, the effect of illumination changes on model performance is an interesting topic. We will focus on this problem in our future research.

## Figures and Tables

**Table 1:** The division scale and number of the frustum.

Scale	Num A	Num B
*T*	1	*T*-1
*T*/2	1	*T*/2-1
*T*/4	1	*T*/4-1
*T*/8	1	*T*/8-1

**Table 2:** The difficulty level officially provided by the KITTI dataset.

Level	Min Bounding Box Height	Max Occlusion Level	Max Truncation
Easy	40 Px	Fully visible	15%
Moderate	25 Px	Partly occluded	30%
Hard	25 Px	Difficult to see	50%

**Table 3:** Effects of using different modules.

Backbone	SAF	LFA	PL	Easy	Mod	Hard
Yes				87.95	76.37	68.56
Yes	Yes			87.62 (−0.33)	78.59 (+2.22)	72.74 (+4.18)
Yes		Yes		88.84 (+0.89)	78.11 (+1.74)	71.33 (+2.77)
Yes			Yes	88.91 (+0.96)	77.48 (+1.11)	68.09 (−0.47)
Yes	Yes	Yes		88.71 (+0.76)	79.52(+3.15)	75.69 (+7.13)
Yes	Yes		Yes	88.48 (+0.53)	78.89 (+2.52)	72.81 (+4.25)
Yes		Yes	Yes	89.72 (+1.77)	78.27 (+1.90)	71.17 (+2.61)
Yes	Yes	Yes	Yes	89.46 (+1.51)	79.91 (+3.54)	75.53 (+6.97)

**Table 4:** Performance comparison between our method and the state of the art based on the 2D detector to generate the frustum on the Cars category of the KITTI validation set.

Method	Stage	Number of Parameters	Runtime (s)	AP_3D_ (Cars)			AP_BEV_ (Cars)		
				Easy	Mod	Hard	Easy	Mod	Hard
F-pointnet	Two	-	-	83.76	70.92	63.65	88.16	84.02	76.44
Backbone + Refine	Two	6,633,554	0.49	88.98	78.66	72.23	90.08	88.84	80.10
Backbone	One	3,316,777	0.26	87.95	76.37	68.56	89.88	87.48	78.99
Ours	One	3,724,013	0.29	89.46	79.91	75.53	91.27	89.63	85.75

**Table 5:** Performance comparison between our method and the state of the art based on the 2D detector to generate the frustum on the Pedestrians category of the KITTI validation set.

Method	Stage	Number of Parameters	Runtime (s)	AP_3D_ (Pedestrians)			AP_BEV_ (Pedestrians)		
				Easy	Mod	Hard	Easy	Mod	Hard
F-pointnet	Two	-	-	70.00	61.32	53.59	72.38	66.39	59.57
Backbone + Refine	Two	6,633,554	0.49	70.88	62.24	53.37	72.59	67.05	58.68
Backbone	One	3,316,777	0.26	68.47	60.63	50.80	70.31	66.14	56.09
Ours	One	3,724,013	0.29	70.61	61.84	53.93	72.24	66.58	59.11

**Table 6:** Performance comparison between our method and the state of the art based on the 2D detector to generate the frustum on the Cyclists category of the KITTI validation set.

Method	Stage	Number of Parameters	Runtime (s)	AP_3D_ (Cyclists)			AP_BEV_ (Cyclists)		
				Easy	Mod	Hard	Easy	Mod	Hard
F-pointnet	Two	-	-	77.15	56.49	53.37	81.82	60.03	56.32
Backbone + Refine	Two	6,633,554	0.49	81.69	69.55	59.87	83.28	70.10	61.79
Backbone	One	3,316,777	0.26	75.88	64.63	55.74	80.37	63.24	57.52
Ours	One	3,724,013	0.29	77.24	65.21	56.15	80.79	66.47	57.86

**Table 7:** Performance comparison between our method and the state of the art on the KITTI validation set.

Method	Modality	AP_3D_ (Cars)			AP_BEV_ (Cars)		
		Easy	Mod	Hard	Easy	Mod	Hard
VoxelNet [15]	LiDAR	81.97	65.46	62.85	89.60	84.81	78.57
SECOND [16]	LiDAR	87.43	76.48	69.10	89.96	87.07	79.66
PointRCNN [18]	LiDAR	88.88	78.63	77.38	90.21	87.89	85.51
ContFuse [34]	LiDAR + RGB	86.32	73.25	67.81	95.44	87.34	82.43
AVODFPN [4]	LiDAR + RGB	84.41	74.44	68.65	-	-	-
F-pointnet [8]	LiDAR + RGB	83.76	70.92	63.65	88.16	84.92	76.44
FconvNet [9]	LiDAR + RGB	89.02	78.80	77.09	90.23	88.79	86.84
Ours	LiDAR + RGB	89.46	79.91	75.53	91.27	89.63	85.75

## Data Availability

Not applicable.
